# Synthesis and *in Vitro* Cytotoxic Evaluation of Aminoquinones Structurally Related to Marine Isoquinolinequinones

**DOI:** 10.3390/molecules17067042

**Published:** 2012-06-07

**Authors:** Virginia Delgado, Andrea Ibacache, Cristina Theoduloz, Jaime A. Valderrama

**Affiliations:** 1 Facultad de Química, Pontificia Universidad Católica de Chile, Casilla 306, Santiago 6094411, Chile; 2 Facultad de Ciencias de la Salud, Universidad de Talca, Talca 3460000, Chile; 3 Instituto de Etno-Farmacología (IDE), Universidad Arturo Prat, Casilla 121, Iquique 1100000, Chile

**Keywords:** aminoisoquinoline-5,8-quinones, regioselectivity, anticancer, SAR analysis

## Abstract

The synthesis of 4-methoxycarbonyl-3-methylisoquinolinequinone (**1**) and a variety of its substitution products with amino-, alkylamino and halogen groups on the quinone nucleus is reported. The series of 6-, 7- and 6,7-subtituted isoquinolinequinones were evaluated *in vitro* for their cytotoxic activity using the MTT colorimetric method. All the newly synthesized compounds showed moderate to high potency against MRC-5 healthy lung fibroblasts and four human tumor cell lines: AGS gastric adenocarcinoma, SK-MES-1 lung, J82 bladder carcinoma, and HL-60 leukemia cells. Among the series, compounds **4b**, **12** and **13** exhibited interesting antitumor activity against human gastric adenocarcinoma, human lung and human bladder carcinoma cancer cells. 7-Amino-6-bromoisoquinoline-5,8-quinone (**13**) was found to be the most promising active compound against the tested cancer cell lines, with IC_50_ values in the 0.21−0.49 μM range, lower than the anti-cancer agent etoposide used as reference.

## 1. Introduction

**Figure 1:**
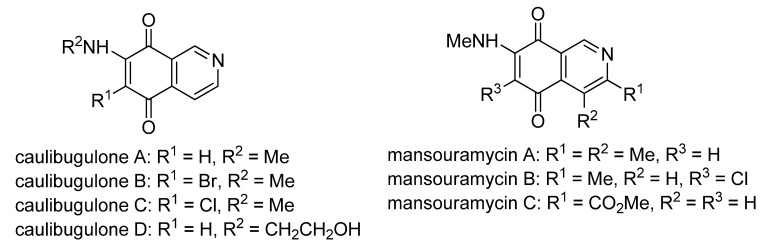
Structure of caulibugulones A–D and mansouramycins A–C.

The search for biologically active natural products from marine sources continues to be an important scientific field that offers promising opportunities for the development of new compounds endowed with pharmacological properties [1,2,3,4,5,6,7,8]. Among the natural products with a quinoide system isoquinolinequinones form an important class characterized by their varied biological properties. For instance, marine isoquinolinequinones such as caulibugulones A–D and mansouramycins A–C (Figure 1) display a significant antitumor profile in many tumor cell lines [9,10]. Caulibugulones A–D, evaluated for antitumor activity against the murine IC-2^WT^ cell line [9] exhibit high potency. In these assays, the A–C members displayed IC_50_ values ranging from 0.22 to 0.34 μg/mL, whereas caulibugulone D showed less cytotoxic activity (IC_50_ = 1.67 μg/mL). According to these IC_50_ values against the tested cell line it can be deduced that insertion of bromine or chlorine atoms at the 6-position in caulibugulone A (0.34 μg/mL), as in caulibugulone B (0.22 μg/mL) and C (0.28 μg/mL), is not an important determinant of cytotoxicity. Furthermore, the replacement of the methylamino group in caulibugulone A by the 2-hydroxyethylamino group, as in caulibugulone D, produces a decrease of the cytotoxic activity. Evidences reported by Wipf and Lazo [11,12] indicate that caulibugulones are selective *in vitro* inhibitors of the Cdc25 family of cell cycle-controlling protein phosphatases. 

The access to caulibugulones A–D via regioselective nucleophilic amination substitution at the 7-position in the reaction of isoquinoline-5,8-quinone with methylamine or 2-aminoethanol in ethanol as solvent in the presence of CeCl_3_·7H_2_O has been simultaneously reported by Wipf [11] and Tamagnan [13]. These amination reactions yield caulibugulones A and D accompanied by low amounts of their corresponding 6-amino-substituted regioisomers. Further reaction of caulibugulone A with *N*-chlorosuccinimide and *N*-bromosuccinimide provides access to caulibugulones B and C, respectively.

Regarding mansouramycins A–C, screening on a panel of up to 36 human tumor cell lines displayed significant cytotoxicity, with pronounced selectivity for non-small cell lung cancer, breast cancer, melanoma, and prostate cancer cells [10]. Mansouramycin C (**4**) proved to be the most active compound, with an overall potency of 0.089 μM. It was followed by mansouramycin B (mean IC_50_ = 2.7 μM) and then mansouramycin A, with a mean IC_50_ value of 13.44 μM. These data indicate that insertion of a methoxycarbonyl group at C-1 or a chlorine atom at C-6 into the 7-methylaminoisoquinolinequinone pharmacophore induces an increase of antitumor activity. 

As part of our research program aimed at the synthesis and structure-activity relationship (SAR) of novel aminoquinones as potential antitumor agents [14,15,16,17,18] we report herein the synthesis and the *in vitro* antitumor evaluation of a variety of amino-, alkylamino and alkylamino-haloisoquinolinequinone derivatives, structurally related with the above mentioned marine isoquinolinequinones, as well the SAR of this series.

## 2. Results and Discussion

**Scheme 1:**
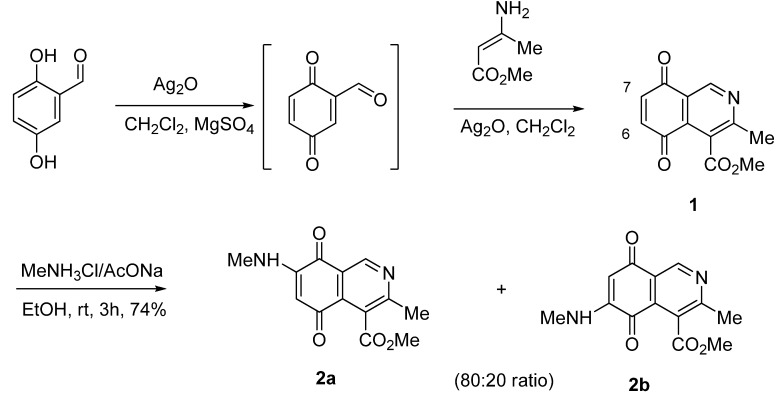
Synthesis and reaction of isoquinolinequinone **1** with methylamine.

Isoquinolinequinone **1**, unsubstituted at the 1-position and containing a methoxycarbonyl group on the heterocyclic ring, as in mansouramycin C, was selected as a suitable precursor of the designed aminoisoquinolinequinones. The synthesis of the target compound **1** was accomplished in 86% yield from commercially available 2,5-dihydroxybenzaldehyde and methyl aminocrotonate, through a one-pot procedure previously developed in our laboratory [19] (Scheme 1).

**Scheme 2:**
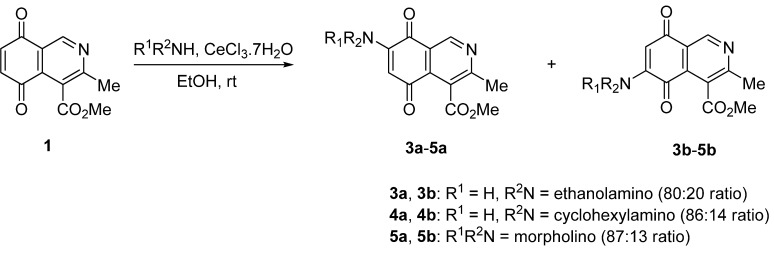
Reaction of isoquinolinequinone **1** with amines induced by acid-catalysis.

The amination reaction of quinone **1** was firstly examined with methylamine hydrochloride-sodium acetate in ethanol at room temperature. The reaction went to completion in 3 h to give an 80:20 mixture of regioisomers **2a** and **2b** (as evaluated by ^1^H-NMR) in 74% yield. Further separation by column chromatography on silica gel afforded pure compound **2a** in 52% yield (Scheme 1). Our efforts to isolate a pure sample of regioisomer **2b** by chromatography were unsuccessful. Then we examined the amination reaction of quinone **1** with 2-aminoethanol under the same conditions used with methylamine. Analysis of the progress of the reaction by TLC reveals that the amination reaction of **1** proceeds slowly (>7 h) and extensive decomposition of quinone **1** was detected. According to precedents on the use of a Lewis acid to facilitate the reaction of quinones with amines [20,21,22,23,24], compound **1** was allowed to react with 2-aminoethanol in ethanol in the presence of 5 mmol% of CeCl_3_·7H_2_O. Under these conditions, the reaction went to completion in 3 h to give an 80:20 mixture of regioisomers **3a** and **3b** (as evaluated by ^1^H-NMR) in 65% yield. Further separation by column chromatography on silica gel afforded the major regioisomer **3a** pure in 47% yield (Scheme 2). Attempts to isolate a pure sample of the minor regioisomer **3b** by chromatography were unsuccessful.

The amination reactions of quinone **1** with cyclohexylamine and morpholine under the above acid-induced conditions were also explored. The experiments provided access to a mixture of the corresponding alkylaminoisoquinolinequinones **4a**,**b** and **5a**,**b** (Scheme 2). In these cases, pure samples of the regioisomers of each pair were isolated by column chromatography. 

According to the amination reaction of quinone **1** with the studied alkyamines we can conclude that the addition of nitrogen nucleophiles across the quinone double bond occurs with high regioselective preference to give the 7-substituted regioisomer as the main product (Scheme 2). 

**Figure 2:**
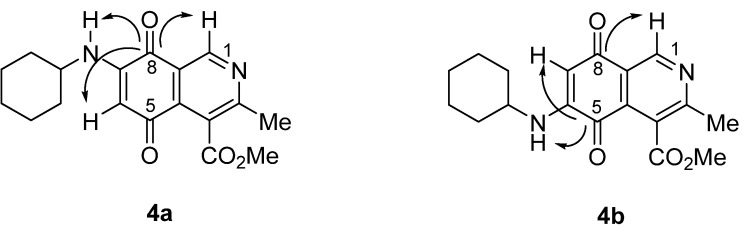
HMBC correlations of regioisomers **4a** and **4b**.

The structure of the new compounds was established on the basis of their nuclear magnetic resonance (^1^H-NMR, ^13^C-NMR, 2D-NMR) and high resolution mass spectra (HRMS). The position of the nitrogen substituent in these aminoquinones was determined by means of HMBC experiments. For example, the location of the nitrogen group at C-7 in compound **4a** was deduced by the ^3^*J*_C,H _couplings between the carbon at C-8 (δ 180.83) with the protons at C-1 (δ 9.17), at C-6 (δ 5.77) and that of the NH group (δ 5.96). In the case of aminoquinone **4b**, the location of the nitrogen substituent at C-6 was established by ^3^*J*_C,H _coupling between the carbon at C-5 (δ 181.71) with the proton at C-7 (δ 5.79) and the proton of the NH group (δ 5.75). Also, the ^3^*J*_C,H_ coupling between the carbon at C-8 (δ 181.49) with the proton at C-1 (δ 9.29) can be observed (Figure 2).

Aminoisoquinolinequinones **2a**, **3a**, **4a** and **5a** were submitted to halogenations with *N*-halosuccinimide to yield the corresponding alkylamino-haloisoquinolinequinones (Table 1).

**Table 1:** Preparation of aminohaloisoquinolinequinones **6**–**11**.

Substrate	Product	N°	Yield (%) *	Substrate	Product	N°	Yield (%) *
**2a**	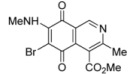	**6**	80	**3a**	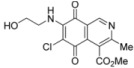	**9**	78
**2a**	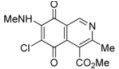	**7**	85	**4a**	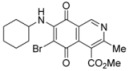	**10**	84
**3a**	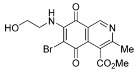	**8**	75	**5a**	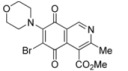	**11**	70

* Isolated yields.

**Scheme 3:**
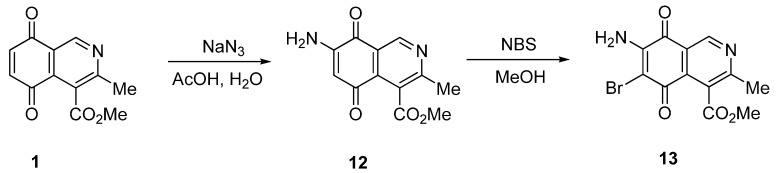
Preparation of amino- and aminobromoisoquinolinequinones **12** and **13**.

We also explored the preparation of amino- and aminobromoisoquinolinequinones in order to include them in the biological assays. Treatment of quinone **1 **with sodium azide in acetic acid, according to a previously reported procedure [15], afforded aminoisoquinolinequinone **12** as the sole regioisomer in 51% yield (Scheme 3). The structure of compound **12 **was established by means of HMBC experiments. Further bromination of **12** with *N*-bromosuccinimide, provides aminobromoisoquinolinequinone **13** in 86% yield.

Isoquinolinequinones **1**, **2a**, **3a**, **4a**, **4b**, **5a**, **6–13** were evaluated for their *in vitro* cytotoxic activity against MRC-5 (healthy lung fibroblasts) and four human cancer cell lines: AGS (gastric), SK-MES-1 (lung), J82 (bladder), and HL-60 (leukemia), using the conventional microculture tetrazolium reduction assay [25,26,27]. The broad variety of the synthesized compounds was designed in order to gain insight into the influence of nitrogen and halogen groups on the quinone nucleus of the isoquinolinequinone pharmacophore on the biological activity. Table 2 summarizes the data from these evaluations. According to the IC_50_ values collected in Table 2, it is evident that insertion of the nitrogen substituents in quinone **1**, as in **2a**,**3a**,**4a**,**4b**,**5a** and **5b**, increases the cytotoxic activity in all the evaluated cell lines, compared to those of precursor **1**. The initial structure-activity relationship (SAR) was focused on the nature and location of alkylamino at the quinone nucleus of the isoquinolinequinone pharmacophore. The data of Table 2 for compounds **2a**, **3a**, **4a** and **5a** reveal that the shape and polarity of the alkylamino group at the 7-position in the isoquinolinequinone moiety does not significantly influence the biological activity. Concerning the effect of the position of the alkylamino groups, the IC_50_ values for the regioisomers **4a/4b** and **5a/5b** indicate that substitution of the nitrogen group at the 6-position of the quinone pharmacophore induces a greater effect on the antitumor activity than at the 7-position. It can be seen that the effect of such substitution on the antitumor potency on gastric adenocarcinoma and lung cancer cells reaches values nearly seven times higher compared to regioisomer **4a**.

**Table 2:** Cytotoxic activity of **1** and their aminoisoquinoline-5,8-quinone derivatives.

		IC_50_ ± SEM ^a^ (μM)				
N°	Structure	MRC-5 ^b^	AGS ^c^	SK-MES1 ^d^	J82 ^e^	HL-60 ^f^
**1**	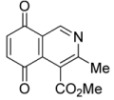	4.5 ± 0.3	17.34 ± 1.64	25.9 ± 1.6	14.81 ± 0.74	14.81 ± 0.74
**2a**	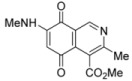	8.1 ± 0.3	3.5 ± 0.2	5.22 ± 0.38	5.1 ± 0.4	9.74 ± 0.76
**3a**	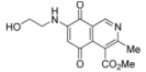	7.9 ± 0.3	2.3 ± 0.1	5.99 ± 0.36	7.2 ± 0.4	6.16 ± 0.36
**4a**	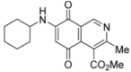	10.1 ± 0.6	4.0 ± 0.2	5.33 ± 0.26	12.0 ± 1.0	10.67 ± 0.64
**4b**	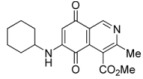	2.2 ± 0.1	0.59 ± 0.03	1.69 ± 0.09	3.0 ± 0.2	1.57 ± 0.13
**5a**	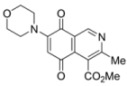	6.7 ± 0.4	1.75 ± 0.09	4.00 ± 0.22	8.56 ± 0.27	2.99 ± 0.15
**5b**	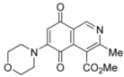	3.77 ± 0.21	1.20 ± 0.06	1.76 ± 0.11	2.99 ± 0.12	2.11 ± 0.13
**6**	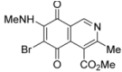	2.6 ± 0.2	1.8 ± 0.1	1.66 ± 0.08	2.0 ± 0.1	2.64 ± 0.21
**7**	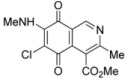	1.9 ± 0.2	1.9 ± 0.0	1.15 ± 0.05	1.2 ± 0.1	2.63 ± 0.13
**8**	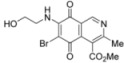	2.7 ± 0.2	1.1 ± 0.0	2.13 ± 0.13	2.2 ± 0.2	3.57 ± 0.16
**9**	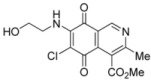	7.1 ± 0.5	2.6 ± 0.2	1.29 ± 0.08	1.2 ± 0.1	2.34 ± 0.14
**10**	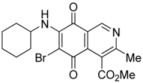	2.2 ± 0.2	0.56 ± 0.03	2.14 ± 0.13	2.3 ± 0.1	2.15 ± 0.12
**11**	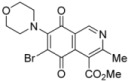	9.53 ± 0.38	8.15 ± 0.49	1.46 ± 0.06	6.97 ± 0.35	2.61 ± 0.18
**12**	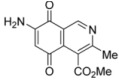	1.8 ± 0.1	1.2 ± 0.1	0.93 ± 0.03	1.1 ± 0.1	2.16 ± 0.09
**13**	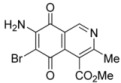	0.28 ± 0.01	0.26 ± 0.01	0.21 ± 0.01	0.29 ± 0.01	0.49 ± 0.02
	Etoposide	0.33 ± 0.02	0.58 ± 0.02	1.83 ± 0.09	3.49 ± 0.16	2.23 ± 0.09

^a^ Data represent mean average values for six independent determinations; ^b^ Normal human lung fibroblasts cells; ^c^Human gastric adenocarcinoma cell line; ^d^ Human lung cancer cell line; ^e^ Human bladder carcinoma cell line; ^f^ Leukemia cell line.

Next, the SAR analysis was focused on the effects of insertion of chlorine and bromine atoms at the 6-position of the 7-substituted alkylaminoisoquinolinequinones **2a**, **3a**, **4a** and **5a**. Comparison of the IC_50_ values of halo derivatives **6**–**11** with those of their corresponding precursors indicates that the substitution of halogen atom at the 6-position enhances the antitumor potency in all the evaluated cell lines, except for compound **11** on lung fibroblasts and gastric cancer cells. 

According to the data of Table 2, aminoisoquinolinequinone **12** showed more cytotoxic activity (0.93–2.16 µM) on the cancer cell lines than the *N*-alkyl 7-substituted analogs **2a**, **3a**, **4a** and **5a**. It is noteworthy that compound **12** exhibits higher antitumor potency on lung and bladder cell lines than the anticancer drug etoposide.

Comparison of the cytotoxic potency of compound **12** (0.93−2.16 µM) with that of its bromoderivative **13** (IC_50_: 0.21−0.49 µM) reveals that insertion of the bromine atom at the 6-position of **12** induces a high increase of the cytotoxic activity in all the evaluated cell lines. It is noteworthy that compounds **12 **and **13** exhibit higher antitumor potency and selectivity index (SI = IC_50_ fibroblasts/IC_50_ cancer cells. **12**: 0.8–1.9; **13**: 0.5–1.3) in almost all cell lines than the anticancer drug etoposide (SI: 0.1–0.6). Among the tested analogs of the series, compounds **4b**, **12** and **13** are the most significant antitumor members. Compound **13** was selected as a promising lead compound due to its high potency on the tested tumor cell lines and selectivity index.

## 3. Experimental

### 3.1. General

All reagents were commercially available reagent grade and were used without further purification. Melting points were determined on a Stuart Scientific SMP3 apparatus and are uncorrected. ^1^H-NMR spectra were recorded on Bruker AM-200 and Avance-400 instruments in deuterochloroform (CDCl_3_). ^13^C-NMR spectra were obtained in CDCl_3_ at 50 and 100 MHz. Bidimensional NMR techniques and DEPT were used for signal assignment. Chemical shifts are expressed in ppm downfield relative to tetramethylsilane and the coupling constants (*J*) are reported in Hertz. The HRMS spectra were obtained on a Thermo Finnigan spectrometer, model MAT 95XP. Silica gel Merck 60 (70–230 mesh) was used for preparative column chromatography and TLC aluminum foil 60F_254_ for analytical TLC.

### 3.2. Chemistry

*4-Methoxycarbonyl-3-methylisoquinoline-5,8-quinone *(**1**). A suspension of 2,5-dihydroxybenzaldehyde (1 mmol), methyl 3-aminocrotonate (1 mmol), Ag_2_O (2 mmol) and MgSO_4_ (0.5 g) in CH_2_Cl_2_ (25 mL) was stirred at rt for 3 h. The mixture was filtered, the solids were washed with CH_2_Cl_2_ and the solvent removed under reduced pressure. The residue was column cromatographed over silica gel (90:10 CH_2_Cl_2_/AcOEt) to yield pure quinone **1** (86%) as yellow solid, mp 109–111.5 °C; IR ν_max_ 1731 (C=O ester), 1668 (C=O quinone); ^1^H-NMR (400 MHz, CDCl_3_): δ 2.65 (s, 3H, Me), 4.03 (s, 3H, CO_2_Me), 7.04 (s, 2H, 6- and 7-H), 9.22 (s, 1H, 1-H); ^13^C-NMR (100 MHz): δ 22.69, 53.11, 122.18, 125.15, 133.39, 138.38, 138.58, 148.57, 161.83, 167.68, 183.38, 183.60; HRMS (M^+^): *m/z* calcd for C_12_H_9_NO_4_: 231.05315; found: 231.05229.

*7-Methylamino-4-methoxycarbonyl-3-methylisoquinoline-5,8-quinone *(**2a**). A suspension of quinone **1** (1 mmol), methylamine hydrochloride (2 mmol) and AcONa (2 mmol) in ethanol (20 mL) was stirred at rt for 1.5 h. The solvent was removed under reduced pressure to give an orange solid containing regioisomers **2a** and **2b** (74%) in 80:20 proportion. The major regioisomer **2a** was isolated by column chromatography over silica gel (90:10 CH_2_Cl_2_/AcOEt): red solid (52 %), mp 209.5–211 °C; IR ν_max_ 3279 (N-H), 1740 (C=O ester), 1681 (C=O quinone); ^1^H-NMR: (400 MHz, DMSO): δ 2.66 (s, 3H, Me), 2.95 (d , 3H, *J* = 5.4 Hz, NHMe), 4.04 (s, 3H, CO_2_Me), 5.75 (s, 1H, 6-H), 6.09 (br s, 1H, NH), 9.18 (s, 1H, 1-H); ^13^C-NMR (100 MHz) δ: 23.02, 29.36, 53.21, 101.16, 122.08, 126.38, 136.38, 148.10, 148.82, 163.02, 168.94, 180.24, 180.55; HRMS (M^+^): *m/z* calcd for C_13_H_12_N_2_O_4_: 260.0797; found: 260.0731.

*7-(2-Hydroxyethylamino)-4-methoxycarbonyl-3-methylisoquinoline-5,8-quinone *(**3a**). A suspension of quinone **1** (1 mmol), 2-aminoethanol (2 mmol), CeCl_3_.7H_2_O (0.05 mmol) and ethanol (25 mL) was left with stirring at rt for 3 h. The solvent was removed under reduced pressure to give an orange solid containing regioisomers **3a** and **3b** (65%) in 80:20 proportion. The major regioisomer **3a** was isolated by column chromatography over silica gel (90:10 CH_2_Cl_2_/AcOEt); red solid (47%), mp 195.5–197 °C; IR ν_max_ 3399 (O-H), 3186(N-H), 1700 (C=O ester), 1600 (C=O quinone); ^1^H-NMR (400 MHz, DMSO): δ 2.52 (s, 3H, Me), 3.27 (dt, 2H, *J* = 5.8, 11.7 Hz, NHCH_2_), 3.59 (dt, 2H, *J* = 5.6, 11.2 Hz, CH_2_OH), 3.88 (s, 3H, CO_2_Me), 4.89 (t, 1H, *J* = 5.7 Hz, OH), 5.79 (s, 1H, 6-H), 7.75 (t, 1H, *J* = 5.9 Hz, NH), 9.06 (s, 1H, 1-H); ^13^C-NMR (100 MHz): δ 22.35, 44.77, 52.63, 58.47, 99.87, 122.43, 125.18, 135.92, 147.36, 149.04, 161.13, 168.25, 178.91, 180.24; HRMS (M^+^): *m/z* calcd for C_14_H_14_N_2_O_5_: 290.0903; found: 290.0891.

*6- and 7-(Cyclohexylamino)-4-methoxycarbonyl-3-methylisoquinoline-5,8-quinone *(**4a**,**4b**). A suspension of quinone **1** (1 mmol), cyclohexylamine (2 mmol), CeCl_3_·7H_2_O (0.05 mmol) and ethanol (25 mL) was left with stirring at rt for 2 h. The solvent was removed under reduced pressure to give an orange solid containing regioisomers **4a** and **4b** in 86:14 ratio. These compounds were isolated by column chromatography over silica gel (90:10 CH_2_Cl_2_/AcOEt).

Compound **4a **(less polar, 80%): orange solid, mp 161.5–163.5 °C; IR ν_max_ 3,246 (N-H), 2,927 and 2,856 (CH), 1741 (C=O ester), 1685 (C=O quinone); ^1^H-NMR (400 MHz, CDCl_3_): δ 1.35 (m, 5H, CH_2_), 1.68 (m, 1H, CH_2_), 1.81 (m, 2H, CH_2_), 2.03 (m, 2H, CH_2_), 2.65 (s, 3H, Me), 3.30 (m,1H, CH), 4.03 (s, 3H, CO_2_Me), 5.77 (s, 1H, 6-H), 5.96 (d, 1H, *J* = 7.5 Hz, NH), 9.17 (s, 1H, 1-H); ^13^C-NMR (100 MHz): δ 22.99, 24.54 (2C), 25.42, 31.83 (2C), 51.44, 53.16, 101.25, 122.11, 126.23, 136.34, 146.52, 148.03, 162.89, 168.93, 180.13, 180.83; HRMS (M^+^): *m/z* calcd for C_18_H_20_N_2_O_4_: 328.14227; found: 328.14083.

Compound **4b** (9.5%): red solid, mp 158.5–160 °C; IR ν_max_ 3246 (N-H); 2927 and 2856 (CH); 1741 (C=O ester); 1685 (C=O quinone); ^1^H-NMR (400 MHz, CDCl_3_): δ 1.35 (m, 5H, CH_2_), 1.68 (m, 1H, CH_2_), 1.81 (m, 2H, CH_2_), 2.03 (m, 2H, CH_2_), 2.65 (s, 3H, Me), 3.30 (m, 1H, CH), 4.05 (s, 3H, CO_2_Me), 5.75 (d, 1H, *J* = 7.8 Hz, NH), 5.79 (s, 1H, 6-H), 9.28 (s, 1H, 1-H), ^13^C-NMR (100 MHz): δ 22.64, 24.54 (2C), 25.47, 31.86 (2C), 51.39, 53.26, 101.23, 123.05, 124.92, 132.44, 146.31, 148.73, 159.91, 168.51, 181.49, 181.71;HRMS (M^+^): *m/z* calcd for C_18_H_20_N_2_O_4_: 328.14227; found: 328.14070.

*6- and 7-Morpholino-4-methoxycarbonyl-3-methylisoquinoline-5,8-quinone* (**5a**,**5b**). A suspension of quinone **1** (1 mmol), morpholine (2 mmol), CeCl_3_·7H_2_O (0.05 mmol) and ethanol (25 mL) was left with stirring at rt for 2 h. The solvent was removed under reduced pressure to give an orange solid containing regioisomers **5a** and **5b** in 87:13 proportion. These compounds were isolated by column chromatography over silica gel (90:10 CH_2_Cl_2_/AcOEt).

Compound **5a** (less polar, 72%): dark orange solid, mp 169.5–171.5 °C; IR ν_max_ 1722 (C=O ester), 1,696 and 1,638 (C=O quinone); ^1^H-NMR (400 MHz, CDCl_3_): δ 2.65 (s, 3H, Me), 3.59 (t, 4H, *J* = 4.7 Hz, N-CH_2_), 3.87 (t, 4H, *J* = 4.7 Hz, O-CH_2_), 4.03 (s, 3H, CO_2_Me), 6.01 (s, 1H, 6-H), 9.14 (s, 1H, 1-H); ^13^C-NMR (100 MHz): δ 22.90, 49.21 (2C), 53.28, 66.49 (2C), 110.92, 123.98, 125.36, 134.86, 148.72, 152.78, 161.98, 168.72, 181.16, 181.75; HRMS (M^+^): *m/z* calcd for C_16_H_16_N_2_O_5_: 316.10593; found: 316.10521. 

Compound **5b** (17%): dark red solid, mp 159.5–162 °C; IR ν_max_ 1724 (C=O ester), 1,678 and 1,634 (C=O quinone); ^1^H-NMR (400 MHz, CDCl_3_): δ 2.66 (s, 3H, Me), 3.47 (t, 4H, *J* = 4.8, N-CH_2_), 3.85 (t, 4H, *J* = 4.8 Hz, O-CH_2_), 4.03 (s, 3H, CO_2_Me), 6.01 (s, 1H, 6-H), 9.23 (s, 1H, 1-H); ^13^C-NMR (100 MHz): δ 22.89, 49.10 (2C), 53.25, 66.44 (2C), 111.30, 122.43, 125.41, 135.43, 148.35, 153.21, 160.76, 168.54, 182.10, 182.30; HRMS (M^+^): *m/z* calcd for C_16_H_16_N_2_O_5_: 316.10593; found: 316.10445. 

### 3.3. General Procedure for the Synthesis of 6-Bromo (or 6-chloro)-7-aminoisoquinolinequinone Derivatives

A solution of the 7-aminoisoquinolinequinone derivative (1 mmol), the corresponding *N*-bromosuccinimide (NBS) or *N*-chlorosuccinimide (NCS) (1 mmol) and methanol (15 mL) was left with stirring at rt after completion of the reaction as indicated by TLC. The solvent was removed under reduced pressure and the residue was column cromatographed over silica gel (90:10 CH_2_Cl_2_/AcOEt) to yield the corresponding 7-amino-6-haloisoquinolinequinone derivative.

*6-Bromo-4-methoxycarbonyl-7-methylamino-3-methylisoquinoline-5,8-quinone *(**6**). Prepared from **2a** and NBS (30 min, 80%): dark red solid, mp 170–172 °C; IR ν_max_ 3331 (N-H); 1730 (C=O ester), 1687 (C=O quinone); ^1^H-NMR (400 MHz, CDCl_3_): δ 2.65 (s, 3H, Me), 3.48 (d , 3H, *J* = 5.8 Hz, NHMe), 4.05 (s, 3H, CO_2_Me), 6.39 (br s, 1H, NH), 9.16 (s, 1H, 1-H); ^13^C-NMR (100 MHz): δ 23.09, 33.34, 53.39, 121.29, 126.56, 134.72, 146.86, 148.62, 163.23, 168.42, 174.40, 178.84 (the signal of the C-6 was not observed in this experiment); HRMS (M^+^): *m/z* calcd for C_13_H_11_N_2_O_4_Br: 337.99023; found: 337.98951.

*6-Chloro-4-methoxycarbonyl-7-methylamino-3-methylisoquinoline-5,8-quinone *(**7**). Prepared from **2a** and NCS (3 h, 85%): dark red solid, mp 158–160 °C; IR ν_max_ 3331 (N-H); 1773 (C=O ester), 1693 (C=O quinone); ^1^H-NMR (400 MHz, CDCl_3_): δ 2.65 (s, 3H, Me), 3.48 (d , 3H, *J* = 6.0 Hz, NHMe), 4.05 (s, 3H, CO_2_Me), 6.30 (br s, 1H, NH), 9.15 (s, 1H, 1-H); ^13^C-NMR (100 MHz): δ 23.09, 32.82, 53.37, 121.25, 126.52, 135.11, 144.83, 148.50, 163.41, 168.36, 174.83, 179.27 (the signal of the C-6 was not observed in this experiment); HRMS (M^+^): *m/z* calcd for C_13_H_11_N_2_O_4_Cl: 294.0407; found: 294.04002

*6-Bromo-2-(hydroxyethylamino)-4-methoxycarbonyl-3-methylisoquinoline-5,8-quinone *(**8**). Prepared from **3a** and NBS (30 min, 75%): dark red solid, mp 134–136 °C; IR ν_max_ 3463 (O-H), 3320 (N-H), 1720 (C=O ester), 1685 (C=O quinone); ^1^H-NMR (400 MHz, DMSO): δ 2.63 (s, 3H, Me), 3.92 (dt, 2H, *J* = 5.1, 11.2 Hz, CH_2_OH), 4.06 (s, 3H, CO_2_Me), 4.11 (dt, 2H, *J* = 5.3, 10.8 Hz, NHCH_2_), 6.65 (br s, 1H, NH), 9.13 (s, 1H, 1-H); ^13^C-NMR (100 MHz): δ 22.98, 47.25, 53.40, 61.36, 121.49, 126.41, 134.60, 146.84, 148.61, 163.02, 168.52, 174.19, 178.66, (the signal of the C-6 was not observed in this experiment); HRMS (M^+^): *m/z* calcd for C_14_H_13_N_2_O_5_Br: 368.00079; found: 368.00005.

*6-Chloro-2-(hydroxyethylamino)-4-methoxycarbonyl-3-methylisoquinoline-5,8-quinone *(**9**). Prepared from **3a** and NCS (4 h, 78%): dark red solid, mp 123–125 °C; IR ν_max_ 3461 (O-H), 3325 (N-H), 1716 (C=O ester), 1687 (C=O quinone); ^1^H-NMR (400 MHz, CDCl_3_): 1H-NMR (400 MHz, CDCl_3_): δ 2.65 (s, 3H, Me), 3.92 (m, 2H, NHCH_2_), 4.05 (s, 3H, CO_2_Me), 4.07 (m, 2H, CH_2_OH), 6.58 (br s, 1H, NH), 9.16 (s, 1H, 1-H); ^13^C-NMR (100 MHz): δ 23.09, 4.82, 53.42, 61.71, 121.38, 126.48, 134.99, 144.53, 148.59, 163.35, 168.41, 174.67, 179.22, (the signal of the C-6 was not observed in this experiment); HRMS (M^+^): *m/z* calcd for C_14_H_13_N_2_O_5_Cl: 324.0513; found: 324.05073.

*6-Bromo-7-(cyclohexylamino)-4-methoxycarbonyl-3-methylisoquinoline-5,8-quinone *(**10**). Prepared from **4a** and NBS (15 min, 84% yield): dark red solid, mp 132.5–134.5 °C; IR ν_max_ 3312 (N-H), 3008 and 2858 (C-H), 1735 (C=O ester), 1687 (C=O quinone); ^1^H-NMR (400 MHz, CDCl_3_): δ 1.34 (m, 5H, CH_2_), 1.68 (m, 1H, CH_2_), 1.81 (m, 2H, CH_2_), 2.03 (m, 2H, CH_2_), 2.64 (s, 3H, Me), 4.05 (s, 3H, CO_2_Me), 4.58 (m,1H, CH), 6.24 (br s, 1H, NH), 9.15 (s, 1H, 1-H); ^13^C-NMR (100 MHz): δ 23.05, 24.52 (2C), 25.29, 34.50 (2C), 53.32, 61.58, 121.36, 126.59, 134.80, 148.65, 149.99, 163.16, 168.38, 174.32, 178.90, (the signal of the C-6 was not observed in this experiment); HRMS (M^+^): *m/z* calcd for C_18_H_19_N_2_O_4_Br: 406.05280; found: 406.05196.

*6-Bromo-7-morpholino-4-methoxycarbonyl-3-methylisoquinoline-5,8-quinone *(**11**). Prepared from **5a **and NBS (30 min, 70%): violet solid, mp 127.5–129 °C; IR ν_max_ 2955 and 2856 (C-H), 1722 (C=O ester), 1696 and 1638 (C=O quinone); ^1^H-NMR (400 MHz, CDCl_3_): δ 2.66 (s, 3H, Me), 3.70 (t, 4H, *J* = 4.5 Hz, N-CH_2_), 3.90 (t, 4H, *J* = 4.5 Hz, O-CH_2_), 4.05 (s, 3H, CO_2_Me), 9.19 (s, 1H, 1-H); ^13^C-NMR (100 MHz): δ 22.87, 52.59 (2C), 53.42, 67.51 (2C), 115.51, 122.43, 126.05, 133.51, 149.00, 152.08, 162.25, 168.21, 176.25, 180.24; HRMS (M^+^): *m/z* calcd for C_16_H_15_N_2_O_5_Br: 394.01643; found: 394.01457.

*7-Amino-4-methoxycarbonyl-3-methylisoquinoline-5,8-quinone *(**12**). A solution of sodium azide (1.2 mmol) in water (5 mL) was added, with stirring, to a previously heated solution (~40 °C) of quinone **1** (1 mmol) in glacial acetic acid (10 mL). The mixture was stirred at rt for 3.5 h, diluted with water and then extracted with AcOEt (3 × 15 mL). The organic phase was washed with saturated aqueous solution of NaHCO_3_, dried (MgSO_4_) and the solvent was removed under reduced pressure. The residue was column cromatographed over silica gel (80:20 CH_2_Cl_2_/AcOEt) to yield aminoquinone **12** as an orange solid (51%), mp 189–191 °C; IR ν_max_ 3376 and 3140 (N-H), 1732 (C=O ester), 1691 (C=O quinone); ^1^H-NMR (400 MHz, DMSO): δ 2.66 (s, 3H, Me), 4.05 (s, 3H, CO_2_Me), 5.38 (br s, 2H, NH_2_), 6.03 (s, 1H, 6-H), 9.20 (s, 1H, 1-H); ^13^C-NMR (100 MHz): δ 23.37, 52.68, 102.44, 122.47, 125.17, 135.92, 147.19, 150.88, 161.00, 168.34, 179.30, 180.65; HRMS (M^+^): m/z calcd for C_12_H_10_N_2_O_4_: 246.06406; found: 246.06207.

*7-Amino-6-bromo-4-methoxycarbonyl-3-methylisoquinoline-5,8-quinone *(**13**). Prepared from **12** and NBS (20 min, 86%): mp 146–148 °C; IR ν_max_ 3423 and 3326 (N-H), 1726 (C=O ester), 1683 (C=O quinone); ^1^H-NMR (400 MHz, CDCl_3_): δ 2.67 (s, 3H, Me), 4.06 (s, 3H, CO_2_Me), 5.45 (br s, 1H, NH), 6.26 (br s, 1H, NH), 9.22 (s, 1H, 1-H); ^13^C-NMR (100 MHz): δ 23.18, 53.48, 105.07, 121.08, 126.91, 134.81, 147.20, 148.50, 163.58, 168.26, 174.53, 177.24; HRMS (M^+^): m/z calcd for C_12_H_9_N_2_O_4_Br: 323.97455; found: 323.97419.

### 3.4. Anticancer Assay

The cell lines used in this work were obtained from the American Type Culture Collection (ATCC. Manasas, VA, USA). They included MRC-5 normal human lung fibroblasts (CCL-171), AGS human gastric adenocarcinoma cells (CRL-1739), SK-MES-1 human lung cancer cells (HTB-58) and J82 human bladder carcinoma cells (HTB-1). After the arrival of the cells, they were proliferated in the corresponding culture medium as suggested by the ATCC. The cells were stored in medium containing 10% glycerol in liquid nitrogen. The viability of the cells after thawing was higher than 90%, as assessed by trypan blue exclusion test. Cells were sub-cultured once a week and the medium was changed every two days. Cells were grown in the following media: MRC-5, SKMES-1, and J82 in Eagle's minimal essential medium (EMEM) and AGS cells in Ham F-12. The EMEM medium contained 2 mM L-glutamine, 1 mM sodium pyruvate and 1.5 g/L sodium hydrogen carbonate. Ham F-12 was supplemented with 2 mM L-glutamine and 1.5 g/L sodium hydrogen carbonate. All media were supplemented with 10% heat-inactivated FBS, 100 IU/mL penicillin and 100 μg/mL streptomycin in a humidified incubator with 5% CO_2_ in air at 37 °C. For the experiments, cells were plated at a density of 50,000 cells/mL in 96-well plates. One day after seeding, the cells were treated with the medium containing the compounds at concentrations ranging from 0 up to 100 μM during 3 days and finally the 3-(4,5-dimethylthiazol-2-yl)-2,5-diphenyltetrazolium bromide (MTT) reduction assay was carried out. The final concentration of MTT was 1 mg/mL. The compounds were dissolved in DMSO (1% final concentration) and complete medium. Untreated cells (medium containing 1% DMSO) were used as controls. Each experiment was carried out in sextuplicate.

## 4. Conclusions

**Figure 3:**
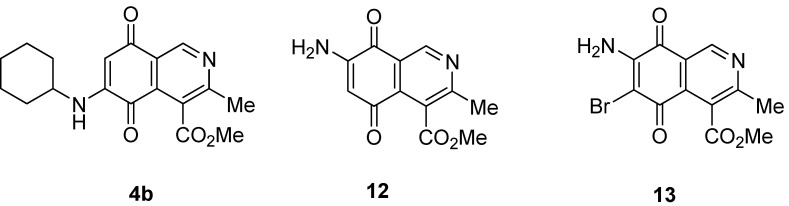
Structures of the isoquinolinequinones selected as the most significant antitumor agents.

We have carried out the synthesis of 4-methoxycarbonyl-3-methylisoquinoline-5,8-quinone (**1**) from 2,5-dihydroxybenzaldehyde, methyl aminocrotonate and silver (I) oxide by employing a highly efficient one-pot procedure. A variety of new isoquinolinequinones substituted with amino, alkylamino and halogen groups were prepared from quinone **1**. The members of this series expressed moderate to high *in vitro* cytotoxic activity against MRC-5 (healthy lung fibroblasts) and four human cancer cell lines: AGS (gastric), SK-MES-1 (lung), J82 (bladder), and HL-60 (leukemia) cell lines. From the current investigation, structure activity relationships of the aminoisoquinolinequinone series demonstrate that nitrogen and halogen substituents at the quinone nucleus of pharmacophore show increased antitumor activity compared with precursor **1**. The effect of such substitutions is more significant in enhancing the antitumor activity for those members containing the cyclohexylamino group at C-6 (compound **4b**), the amino group at C-7 (compound **12**) and the amino and bromine substituents at the 6- and 7-positions (compound **13**). Due to the high incidence of undesirable side-effects induced by the majority of current anticancer drugs and by considering the selectivity index values of aminoquinones **4b** (SI: 0.7–3.7), **12** and **13** (Figure 3), they appear as promising and interesting leads endowed with potential anticancer activity. These results prompt us to design and synthesize more new members of the aminoisoquinoline-5,8-quinones series in order to discover more active and selective anticancer agents. Studies aimed to understand the mechanisms of bioactivity of aminoisoquinolinequinones at cellular level are currently in progress.
